# Multilocus Sequence Typing Reveals Clonality of Fluconazole-Nonsusceptible *Candida tropicalis*: A Study From Wuhan to the Global

**DOI:** 10.3389/fmicb.2020.554249

**Published:** 2020-11-17

**Authors:** Qianyu Wang, Dongling Tang, Kewen Tang, Jing Guo, Yun Huang, Congrong Li

**Affiliations:** ^1^Department of Clinical Laboratory, Renmin Hospital of Wuhan University, Wuhan, China; ^2^Department of Clinical Laboratory, The Third Affiliated Hospital of Zhengzhou University, Zhengzhou, China

**Keywords:** *Candida tropicalis*, azoles, drug resistance, multilocus sequence typing (MLST), epidemiology

## Abstract

*Candida tropicalis* is a globally distributed human pathogenic yeast, and its increasing resistance to azoles makes clinical treatment difficult. In this study, we investigated the clinical features, azole resistance and genetic relatedness of 87 *C. tropicalis* isolates from central China and combined with the global database to explore the relationship between genetic information and fluconazole susceptibility. Of the 55 diploid sequence types (DSTs) identified by multilocus sequence typing (MLST), 27 DSTs were new to the *C. tropicalis* MLST database. Fluconazole-nonsusceptible (FNS) isolates were genetically closely related. goeBURST analysis showed that DST225, DST376, DST506, and DST546 formed a distinct and unique FNS clonal complex (CC) in Wuhan. The local FNS CC belongs to the large FNS CC (CC2) in China, in which the putative founder DST225 has been reported from the environment. The three most prevalent types (DST506, DST525, and DST546) in Wuhan had high minimum inhibitory concentrations (MICs) for antifungal azoles, and the six possible nosocomial transmissions we captured were all FNS strains, most of which were from CC2. Unique FNS CCs have been found in Singapore (CC8) and India (CC17) and are close to China’s CC2 in the minimum spanning tree. There were no FNS CCs outside Asia. This study is the first to reveal a significant correlation between genetic information and fluconazole susceptibility worldwide and to trace geographical locations, which is of great value for molecular epidemiological surveillance and azole-resistance study of *C. tropicalis* globally.

## Introduction

*Candida* species are important components of human normal flora of skin, oral mucosa, vaginal mucosa, and gastrointestinal tract. Due to the increasing number of patients with low immune function and long-term hospitalization, and the widespread use of invasive examination and treatment, *Candida* has become the major opportunistic pathogen causing severe invasive infections, such as candidemia, candidiuria, and pulmonary candidiasis (Falagas et al., 2010; Fesharaki et al., 2013; Dong et al., 2015; Zuza-Alves et al., 2017; Diba et al., 2018). Although *Candida albicans* is the main source of infections, other *Candida* species such as *Candida tropicalis* have been gradually recognized as common yeast pathogens in recent years (Tan et al., 2015; Wu P. F. et al., 2017). In Asia Pacific and Latin America, *C. tropicalis* is ranked as the first or second most prevalent *non-albicans* pathogen (Kumar et al., 2014; Pahwa et al., 2014; Arrua et al., 2015).

*Candida* infections are often treated with azole antifungal drugs. However, with the widespread use of azole agents during the past two decades, azole-resistant and less susceptible clinical isolates have emerged worldwide, especially in the Asia Pacific region (Huang et al., 2014; Fernández-Ruiz et al., 2015; Tan et al., 2016; Fan et al., 2017; Teo et al., 2017; Wu P. F. et al., 2017; Morris et al., 2018). Unfortunately, this phenomenon has brought serious challenges to clinical treatment. Studies in Asia have shown that the non-susceptible rate of *C. tropicalis* to fluconazole is around 20% (mostly cross-resistant with other azoles) and increases rapidly (Wang H. et al., 2016; Fan et al., 2017; Chen et al., 2019). However, fluconazole-nonsusceptible (FNS) *C. tropicalis* is relatively rare in Europe and America (Corzo-Leon et al., 2014; Scordino et al., 2018; Beyer et al., 2019). Some studies support the link between FNS *C. tropicalis* infections and prior antifungal exposure (Choi et al., 2016; Chen et al., 2019), whereas others suggest that FNS *C. tropicalis* may come from the environment (Lo et al., 2017; Wang et al., 2018). Unfortunately, the evolution and geographical distribution of these drug-resistant genotypes remain unclear and require further and more extensive research.

Several molecular typing methods have been widely used in molecular epidemiology of *C. tropicalis*, including multilocus sequence typing (MLST) (Magri et al., 2013; Choi et al., 2016; Fan et al., 2017). This method is based on the single nucleotide polymorphisms (SNPs) analysis of housekeeping gene fragments to distinguish strains and has high discriminatory power and reproducibility (Tavanti et al., 2005). The *C. tropicalis* MLST system recommended by Tavanti et al. has been in use since 2005 and consists of comparisons of six housekeeping genes (*ICL1*, *MDR1*, *SAPT2*, *SAPT4*, *XYR1*, and *ZWF1a*) (Tavanti et al., 2005). As of 24 August 2020, the online database contained data for 1,222 isolates and 939 DSTs (diploid sequence types), including new data obtained in this study^1^. The *C. tropicalis* MLST database enables the comparison of strains and populations from different laboratories and different geographical areas around the world, revealing their different geographical origins, anatomical sources, acquisition and transmission of drug resistance, and high virulence (Zuza-Alves et al., 2017; Wu et al., 2019).

In previous studies on the genetic relationship of FNS *C. tropicalis* isolates, some reported genetic diversity (Magri et al., 2013; Choi et al., 2016; Wu J. Y. et al., 2017), and some proved clonal clustering (Wang Y. et al., 2016; Chew et al., 2017; Fan et al., 2017; Chen et al., 2019). Few of these studies have comprehensively discussed the genetic relationship and geographical distribution, clinical features and patients’ outcomes. We conducted this study to explore these relationships in more detail. We determined the genetic relationships between FS (fluconazole-susceptible) and FNS *C. tropicalis* isolates and analyzed the clinical features and patients’ outcomes based on fluconazole susceptibility and genetic relationship. In conjunction with the global database, we further explored the global geographical distribution, as well as potential evolution and transmission of FS and FNS *C. tropicalis*.

## Materials and Methods

### Clinical Isolates and Microbial Identification

From December 2018 to November 2019, 87 *C. tropicalis* isolates were collected consecutively from the clinical laboratory in Renmin Hospital of Wuhan University (a 5000-bed teaching hospital, more than 5 million outpatients per year), Wuhan, China (Supplementary Table S1). All patients matching the examined isolates presented clinical symptoms suggestive of *Candida* infection. Duplicate isolates, recovered from the same body site of the same patient, were removed. Patients’ medical records were retrospectively analyzed, including demographic data, underlying diseases, infection sites, antifungal therapy, presence of indwelling catheters, fatality, etc. All strains were stored as frozen stocks in 15% glycerol at −80°C. The isolates were subcultured on Sabouraud dextrose agar (SDA) for 24 h at 35°C for identification by matrix-assisted laser desorption/ionization time-of-flight mass spectrometry (Bruker Daltonics, Bremen, Germany), using a recommended standard score ≥1.70 for a correct species-level identification (Lee et al., 2017). The full-tube extraction method using formic acid/acetonitrile (FA/ACN) (Lee et al., 2017; Mizusawa et al., 2017) was used following manufacturer’s instructions.

### Antifungal Susceptibility Testing

The in vitro susceptibility of *C. tropicalis* isolates to fluconazole (FLC), voriconazole (VRC), and itraconazole (ITC) was determined by the broth microdilution method following the M27-E4 standard (CLSI, 2017b) proposed by the Clinical and Laboratory Standards Institute (CLSI). Clinical breakpoints (CBPs) of FLC and VRC were determined at 24 h according to CLSI M60 guideline (CLSI, 2017a) (CBPs to FLC, ≤2 μg/ml for susceptible, ≥8 μg/ml for resistant, and 4 μg/ml for intermediate; CBPs to VRC, ≤0.12 μg/ml for susceptible, ≥1 μg/ml for resistant, and 0.25∼0.5 μg/ml for intermediate). Fluconazole intermediate and resistant types were defined as FNS. WT distribution limit epidemiological cut-off values (ECVs) of ITC were determined at 24 h according to CLSI M59 guideline (≤0.5 μg/ml for wild-type, >0.5 μg/ml for non-wild-type) (CLSI, 2018). Quality control (QC) was ensured by testing *C. parapsilosis* ATCC 22019 and *C. krusei* ATCC 6258. The 24 h MIC QC ranges of *C. parapsilosis* ATCC 22019 (FLC, 0.5∼4 μg/ml; VRC, 0.016∼0.12 μg/ml; ITC, 0.06∼0.5 μg/ml) and *C. krusei* ATCC 6258 (FLC, 8∼64 μg/ml; VRC, 0.06∼0.5 μg/ml; ITC, 0.12∼1 μg/ml) were referred to CLSI M60 (CLSI, 2017a).

### DNA Extraction, PCR Amplification, and Sequencing of *C. tropicalis* Isolates

Whole-genome DNA was extracted from yeast cells using the Yeast Genomic DNA Extraction Kit (Solarbio Life Sciences, Beijing, China). The primers used for amplification and sequencing of six genes have been described previously (Tavanti et al., 2005). Amplification reactions were performed in 25 μl volume containing 12.5 μl 2^∗^Taq PCR PreMix (Innovagene Biotechnology, Changsha, China), 8.5 μl double-distilled H2O, 1.5 μl forward/reverse primers, and 2.5 μl template DNA. Reaction conditions were as follows: 1 cycle of denaturation for 7 min at 94°C, followed by 30 cycles of 94°C for 1 min, 53°C for 1 min and 72°C for 1 min 5 s, with a final extension step of 10 min at 72°C. Following PCR, each product was purified with the PCR Purification Kit (Omega bio-tek, Norcross, America). Bidirectional DNA sequencing was performed by an ABI 3730XL automatic sequencer (Applied Biosystems, Foster, America). Sequencing results were spliced with Geneious 4.8 software^2^ and polymorphic sites were confirmed by visual observation. The heterozygous sites in the chromatograms were defined by the heterozygous data (K,M,R,S,W, and Y) from the International Union of Pure and Applied Chemistry (IUPAC)^3^ nomenclature.

### MLST Analysis of *C. tropicalis*

Allele numbers and DSTs were defined by comparing the sequences with those available in the *C. tropicalis* MLST database^4^. All new alleles and new DSTs were named by the database curator after scrutiny. The MLST data were analyzed with the goeBURST algorithm in PHILOVIZ 2.0 software^5^ to identify clonal complexes (CCs). Isolates were considered the same CC if sharing five out of six alleles. The phylogenetic relationships among the 87 *C. tropicalis* isolates were inferred by using the UPGMA algorithm (unweighted pair group method with arithmetic means) implemented in the MEGA 7 software^6^. Before cluster analysis, DNA sequences were concatenated and then modified to label homozygous and heterozygous sites as described by Tavanti et al. (2005). A bootstrap of 1,000 replications was used during the build process. Bootstrap values of ≥70% were defined as statistically significant (Hillis and Bull, 1993). To assess patterns of evolutionary descent among genotypes based on azole susceptibility and geographical region, the allelic profiles from Wuhan and the global data set (435 isolates with FLC MIC values were included in the analysis) were studied with the goeBURST algorithm in PHILOVIZ 2.0 software and minimal spanning tree algorithm of the BioNumerics 7.6 software^7^.

### Statistical Analysis

Continuous variables were represented by medians and quartile intervals, and categorical variables were represented by absolute frequencies and percentages. To compare clinical factors between FS group and FNS group, and between CC2 FNS group and other FNS group, continuous data were analyzed by Mann-Whitney *U*-test and categorical data were analyzed by χ^2^ or Fisher exact test. The variables were statistically significant only when *P-values* <0.05. All analyses were conducted using SPSS 25.0 software^8^.

## Results

### Description of *C. tropicalis* Isolates and Azole Susceptibility

A total of 87 strains of *C. tropicalis* were isolated from 84 patients during the study period (Supplementary Table S1). Three patients each provided 2 strains (Patient 68: one from blood and one from urine; Patient 1/Patient 7: two strains from urine with an interval of more than 4 months). Among these patients, 41.7% (35/84) were male and 58.3% (49/84) were female. The majority of patients were from the urological ward (14.3%, 12/84) and gynecology ward (14.3%, 12/84), followed by reproductive ward (13.1%, 11/84) and intensive care unit (ICU) ward (10.7%, 9/84), pancreatic surgery ward (7.1%, 6/84), respiratory intensive care unit (RICU) ward (7.1%, 6/84), and other wards (33.4%, 28/84). The isolates were mainly from urine (49.4%, 43/87) and vaginal swabs (25.3%, 22/87), followed by blood (12.6%, 11/87), bile (4.6%, 4/87), sputum (4.6%, 4/87), catheter tips (2.3%, 2/87), and ascites (1.2%, 1/87). A total of 16 patients died in the hospital, from ICU ward (6), RICU ward (5), oncology ward (2), geriatric ward (2), and neurology ward (1), with 10 cases of candidiuria, 4 cases of candidemia (Patient 68 also with candidiuria) and 2 cases of pulmonary candidiasis. The azole susceptibility results of 87 *C. tropicalis* isolates are shown in Table 1. Among 87 clinical *C. tropicalis*, 36 strains were resistant to fluconazole (33 strains were resistant to all tested azoles), 49 strains were susceptible to fluconazole, and 2 strains were intermediate. The FNS rate was 43.7%.

**TABLE 1:** Azole susceptibility results of 87 *C. tropicalis* isolates from Wuhan.

Azole agents	Range (μg/mL)	MIC (μg/mL)		Category (%)			CPBs (μg/mL)			ECV (μg/mL)
			
		50%	90%	S/WT	I	R/NWT	S	I	R	
FLC	0.125∼>64	1	>64	56.3	2.3	41.4	≤2	4	≥8	
VRC	<0.0313∼>16	0.25	16	44.8	13.8	41.4	≤0.125	0.25∼0.5	≥1	
ITC	0.0625∼>16	0.5	16	58.6		41.4				0.5

*S, susceptible; I, intermediate; R, resistant; WT, wild-type; NWT, non-wild-type.*

### *Candida tropicalis* Strain Differentiation by MLST

The size of the sequenced DNA fragments varied from 370 to 525 bp. A total of 152 cases of heterozygosity were detected: *Y* = C + T (44.1%, 67/152), *R* = A + G (27.6%, 42/152), *W* = A + T (14.5%, 22/152), *K* = G + T (7.2%, 11/152), *M* = A + C (4.6%, 7/152), and *S* = G + C (2.0%, 3/152). In the six analyzed gene fragments, 147 (5.5%) polymorphic sites were identified, including 16 in *ICL1*, 26 in *MDR1*, 41 in *SAPT2*, 13 in *SAPT4*, 31 in *XYR1*, and 20 in *ZWF1a* (Table 2). However, some alleles of the *MDR1*, *SAPT4*, and *XYR1* genes showed a different set of heterozygosities at the same nucleotide location. The *SAPT2* gene showed the highest number of polymorphic sites (*n* = 41), while the *SAPT4* locus displayed the lowest (*n* = 13). The *SAPT4* gene presented the highest typing efficiency, distinguishing 1.54 genotypes per polymorphism, whereas *SAPT2* presented the lowest efficiency, distinguishing 0.22 genotypes per polymorphism.

**TABLE 2:** Characteristics of the six MLST housekeeping loci used in this study.

Gene fragment	Heterozygosity						No. of heterozygotes	No. of polymorphic sites	No. of genotypes	Ratio of genotypes to polymorphism
	
	Y	R	W	K	M	S				
ICL1	8	6	1	1	0	0	16	16	7	0.44
MDR1	10	8	6	2	2	1	29	26	24	0.92
SAPT2	17	12	9	2	0	1	41	41	9	0.22
SAPT4	5	3	1	3	1	1	14	13	20	1.54
XYR1	16	8	5	1	2	0	32	31	29	0.94
ZWF1a	11	5	0	2	2	0	20	20	19	0.95
Total	67	42	22	11	7	3	152	147	108	NA

*NA, not applicable.*

DNA sequences from the coding regions of six housekeeping genes were concatenated to obtain a dataset of 2,677 bp for each isolate. The concatenated sequences of 87 isolates were classified into 55 DSTs, with 27 (49.1%) newly identified DSTs (DST976∼DST1002). Fourteen new alleles were identified: 1 in *SAPT2* (allele 62), 3 in *SAPT4* (alleles 107, 108, and 109), 5 in *XYR1* (alleles 167, 168, 169, 170, and 171), and 5 in *ZWF1a* (alleles 61, 62, 63, 64, and 65). All new DSTs and alleles have been submitted to the *C. tropicalis* MLST database. Among the 55 DSTs, no genotype was found to be related to the source of samples. DST506 (*n* = 7), DST525 (*n* = 5), and DST546 (*n* = 5) were the most prevalent. Interestingly, all of these isolates were highly resistant to azole antifungal agents.

### Phylogenetic Analysis and Nosocomial Infection Surveillance

**FIGURE 1:**
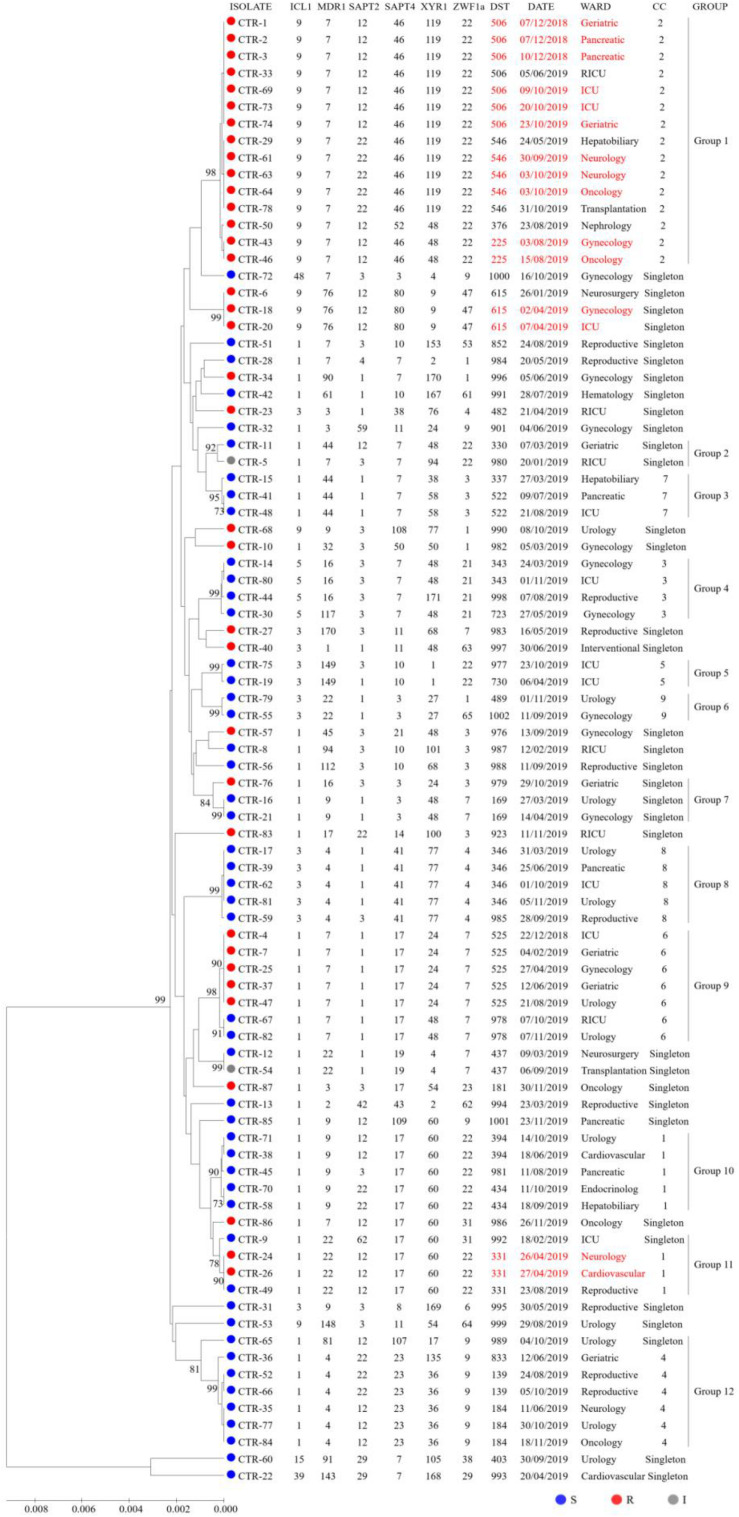
UPGMA dendrogram baseed on MLST date for 87 C. tropicalis isolates from Wuhan. Circles: fluconazole susceptible (blue), resistant (red), or intermediate (gray). The font in red indicates possible nosocomial transmission.

To explore associations among the DSTs and azole susceptibility of the 87 isolates, an unrooted dendrogram was constructed by MEGA 7 software based on UPGMA to evaluate the genetic distance among the isolates. The dendrogram (Figure 1) revealed 12 groups (1∼12) and singletons. Groups were defined by bootstrap values of ≥70% (Hillis and Bull, 1993). goeBURST analysis showed that 24 DSTs were divided into 9 CCs, 31 DSTs were classified as singletons. The CCs measured by goeBURST were consistent with groups defined by UPGMA. CC2 was the most common (15/87, 17.2%) and was 100% similar to UPGMA group 1. According to goeBURST algorithm, CC2 contained 4 DSTs, among which DST225 (*n* = 2) was the putative founder. CC2 was the only FNS aggregation cluster in Wuhan, and the strains all had high MIC to fluconazole and were cross-resistant to voriconazole and itraconazole. Among the FNS isolates from this study, 39.5% (15/38) belonged to CC2, including DST506 (*n* = 5), DST546 (*n* = 7), DST225 (*n* = 2), and DST376 (*n* = 1). The other 23 FNS isolates were scattered among CC1, CC6, and singletons. All strains of CC3, CC4, CC5, CC7, CC8, and CC9 were susceptible to fluconazole.

It is important to note that some patients sharing the same DST may be evidence of nosocomial transmission when analyzing data with epidemiological findings. As can be seen from Figure 1, we captured six possible nosocomial transmissions. Shockingly, all of these infections were caused by FNS *C. tropicalis*, four of which were caused by CC2 strains. In December 2018, isolates from two patients (P2 and P3) in pancreatic surgery ward shared DST506, and around October 2019, isolates from two patients (P60 and P62) in neurology ward shared DST546, highly suggestive of ward spread. During the two times of transmission in the same ward, there were also the same strains isolated from other wards. In addition, the strains of DST615 and DST331 in April 2019, DST225 in August 2019, and DST506 (except for two strains isolated from different specimens of P68 in ICU ward) in October 2019 all came from different wards, but the overlapping length of stay of these patients indicated possible transmission.

### Relationships Between Azole-Resistance, Genetic Evolution, and Geographical Distribution

**FIGURE 2:**
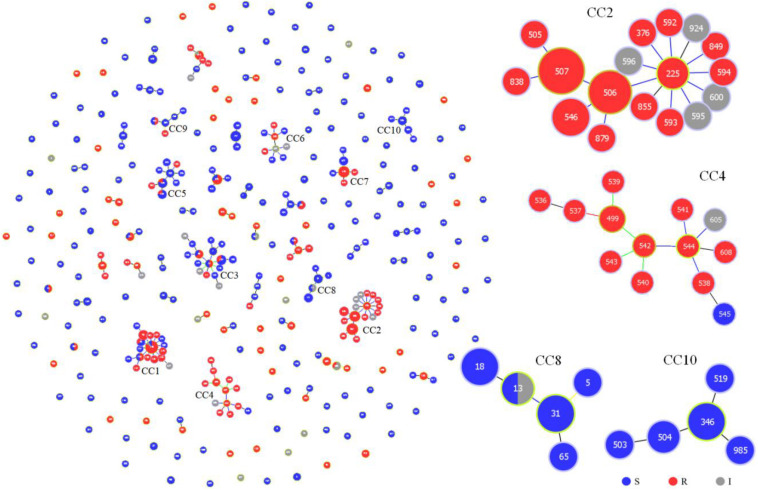
Genetic population structure of 522 *C. tropicalis* isolates. Each circle corresponds to a DST. The size of the circle indicates the number of isolates belonging to the DST and classified as fluconazole susceptible (blue), resistant (red), or intermediate (gray).

We further evaluated the genetic relationships of 87 local clinical isolates with 435 strains available in the MLST database. Of the 522 strains, goeBURST analysis grouped 352 DSTs into 45 CCs and 160 singletons (Figure 2). Twenty-five CCs (1∼25) each contained no less than 3 DSTs. FNS rates of 8 CCs (1, 2, 4, 11, 14, 17, 21, and 24) were all higher than 80%. CC1, CC2, and CC4 with more DSTs had FNS rates as high as 80.8, 100, and 92.9%, respectively. FNS rates of CC17, CC21, and CC24 were all 100%. CC2 was centered on DST225 and contained a total of 17 DSTs (42 strains). The 4 aggregation FNS DSTs in Wuhan all belonged to CC2. CC4 DSTs were not found in Wuhan and only DST181 belonged to CC1. There were also FS CCs. FS rates of CC8 (91.7%), CC10 (100%), CC16 (100%), CC19 (100%), CC22 (100%), CC23 (100%), and CC25 (100%) were all higher than 90% (Supplementary Table S2).

**FIGURE 3:**
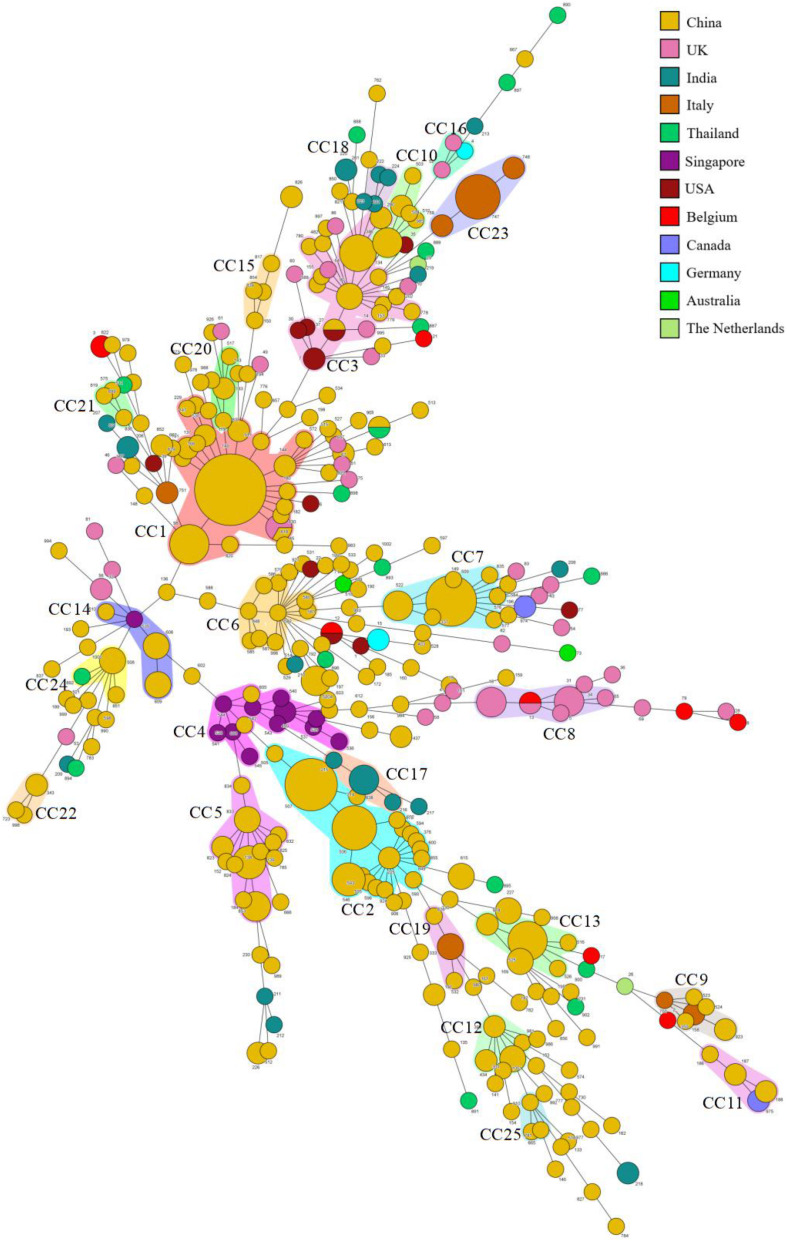
Minimum spanning tree of 522 *C. tropicalis* isolates. Each circle corresponds to a DST. The size of the circle indicates the number of isolates belonging to a specific DST, and the color of the circle represents the country to which it belongs. Shaded areas indicate groups of target clonal complexes (CCs).

We summarized the isolation year, country, region, source, and fluconazole MIC of *C. tropicalis*. Most strains (370/522, 70.9%) were from China. It can be observed in the minimum spanning tree (Figure 3) that only 5 CCs (CC8, CC16, CC17, CC18, and CC23) of the 25 CCs (CC1∼CC25) did not contain strains from China. Eleven of the 25 CCs contained strains from Wuhan. CC12 and CC22 were all from Wuhan. Among the 27 new DSTs discovered in Wuhan, 22 DSTs were not in the 25 CCs and were scattered, indicating that the genetic background of local new DSTs was different from that of other countries and regions. FNS CC1 has been prevalent in Taiwan since 1999, containing 17 DSTs. FNS CC2, CC4, and CC17 were from China, Singapore (except DST605 and DST608 came from Nanchang, China), and India, respectively, and all strains were after 2011. The genetic distances of CC2, CC4, and CC17 are relatively close, indicating that FNS strains from the three Asian countries may have similar genetic backgrounds. The putative founder DST225 in CC2 was isolated from the environment and hospitals in Taiwan, and also was isolated in hospitals from the Chinese mainland (Beijing and Wuhan) (Supplementary Table S3). We found no FNS CCs outside Asia. Strains of FS CC8 (United Kingdom:11, Belgium:1), CC16 (United Kingdom:2, Germany:1), and CC23 (Italy:13) were all from European countries. The FS rates in this study from Australia, Belgium, Canada, Germany, Italy, United Kingdom, United States, and the Netherlands were 100% (2/2), 77.8% (7/9), 75.0% (3/4), 66.7% (2/3), 100% (21/21), 88.6% (39/44), 91.7% (11/12), and 50.0% (1/2), respectively. The FS rates of China, India, Singapore, and Thailand were 49.5% (183/370), 24.0% (6/25), 7.7% (1/13), and 70.6% (12/17), respectively. Strains from Europe, America, and Oceania were generally more susceptible to fluconazole than those from Asia.

### Clinical Features of Patients With *C. tropicalis*

We analyzed the demographics, comorbid conditions, healthcare factors, wards, primary infection sites, and outcomes of 84 patients. Table 3 shows that heart disease (*P* = 0.03), antifungal drug exposure (*P* = 0.03), and hospitalization in medical wards (*P* = 0.02) were associated with FNS *C. tropicalis* infection. There was no statistically significant difference in any factor of patients infected with FNS CC2 and other FNS *C. tropicalis*. We found no significant difference in deaths between FS group and FNS group, or between FNS CC2 group and other FNS group.

**TABLE 3:** Comparisons of clinical and microbiological characteristics between FS and FNS, FNS CC2 and Other FNS *C. tropicalis* infections, Wuhan.

Characteristic	FS Infection (*n* = 49)	FNS Infection (*n* = 35)	*P*-value	FNS CC2 Infection (*n* = 13)	Other FNS Infection (*n* = 22)	*P*-value
**Demographics**						
Age, *y*, median (IQR)	55.0 (37.0–67.0)	57.0 (44.5–76.5)	0.19	63.0 (56.0–82.0)	55.0 (44.3–73.8)	0.35
Sex *n* (%)			0.48			0.72
M	22 (44.9)	13 (37.1)		4 (30.8)	9 (40.9)	
F	27 (55.1)	22 (62.9)		9 (69.2)	13 (59.1)	
**Comorbid conditions^a^*n* (%)**						
Heart disease	9 (18.4)	14 (40.0)	0.03*	4 (30.8)	10 (45.5)	0.49
Lung diseases	16 (32.7)	17 (48.6)	0.14	9 (69.2)	8 (36.4)	0.09
Hepatobiliary/pancreatic diseases	10 (20.4)	10 (28.6)	0.39	4 (30.8)	6 (27.3)	1.00
Kidney disease	10 (20.4)	13 (37.1)	0.09	6 (46.2)	7 (31.8)	0.48
Brain diseases	9 (18.4)	11 (31.4)	0.17	4 (30.8)	7 (31.8)	1.00
Solid tumor	4 (8.2)	7 (20.0)	0.21	4 (30.8)	3 (13.6)	0.38
Diabetes	9 (18.4)	4 (11.4)	0.39	1 (7.7)	3 (13.6)	1.00
Hypertension	13 (26.5)	14 (40.0)	0.19	6 (46.2)	8 (36.4)	0.72
Gynecological disease	15 (30.6)	6 (17.1)	0.16	2 (15.4)	4 (18.2)	1.00
**Healthcare factors *n* (%)**						
Solid-organ transplant	1 (2.0)	0	1.00	0	0	–
Surgery	12 (24.5)	9 (25.7)	0.90	5 (38.5)	4 (18.2)	0.24
Mechanical ventilator	13 (26.5)	14 (40.0)	0.19	7 (53.8)	7 (31.8)	0.29
Indwelling urinary catheter	18 (36.7)	18 (51.4)	0.18	8 (61.5)	10 (45.5)	0.49
Central venous catheter	12 (24.5)	13 (37.1)	0.21	6 (46.2)	7 (31.8)	0.48
Antifungal drug exposure	6 (12.2)	11 (31.4)	0.03*	4 (30.8)	7 (31.8)	1.00
Antimicrobial drug exposure	38 (77.6)	28 (80.0)	0.79	11 (84.6)	17 (77.3)	0.69
**Primary infection sites *n* (%)**						
Bloodstream infection	7 (14.3)	6 (17.1)	0.72	2 (15.4)	4 (18.2)	1.00
Visceral infection	3 (6.1)	2 (5.7)	1.00	1 (7.7)	1 (4.5)	1.00
Urinary tract infection	20 (40.8)	20 (57.2)	0.14	8 (61.5)	12 (54.6)	0.74
Respiratory tract infection	3 (6.1)	1 (2.9)	0.86	1 (7.7)	0	0.37
Reproductive tract infection	16 (32.7)	6 (17.1)	0.11	1 (7.7)	5 (22.7)	0.38
**Wards *n* (%)**						
ICU/RICU	8 (16.3)	7 (20.0)	0.67	2 (15.4)	5 (22.7)	0.69
Gynecology/Reproductive	16 (32.7)	7 (20.0)	0.20	1 (7.7)	6 (27.3)	0.22
Surgery^b^	19 (38.8)	9 (25.7)	0.21	4 (30.8)	5 (22.7)	0.70
Medical^c^	6 (12.2)	12 (34.3)	0.02*	6 (46.1)	6 (27.3)	0.29
**Death *n* (%)**						
All	6 (12.2)	10 (28.6)	0.06	3 (23.1)	7 (31.8)	0.71
With pulmonary candidiasis^d^	1 (33.3)	1 (100.0)	1.00	1 (100.0)	0	−
With candidemia^e^	1 (14.3)	3 (50.0)	0.27	1 (50.0)	2 (50.0)	1.00
With candidiuria^f^	4 (20.0)	6 (30.0)	0.47	1 (12.5)	5 (41.7)	0.33

*^*a*^All systemic diseases were not graded, including mild-severe infection, mild organ dysfunction-severe organ failure.*

*^*b*^Surgery wards: including Urology ward, Pancreatic ward, Hepatobiliary ward, Cardiovascular Surgery ward, Neurosurgery ward, Transplantation ward, and Interventional ward.*

*^*c*^Medical wards: including Geriatric ward, Oncology ward, neurology ward, Cardiovascular Medicine ward, Endocrinology ward, Hematology ward, and Nephrology ward.*

*^*d*^With pulmonary candidiasis: A total of 4 cases. FS Infection (*n* = 3), FNS Infection (*n* = 1); FNS CC2 Infection (*n* = 1), Other FNS Infection (*n* = 0). There was only 1 case of pulmonary Candida infection with FNS *C. tropicalis* (in CC2), which could not be compared in groups (FNS CC2 Infection group and Other FNS Infection group), so the *P-*value was shown as “−”.*

*^*e*^With candidemia: A total of 13 cases. FS Infection (*n* = 7), FNS Infection (*n* = 6); FNS CC2 Infection (*n* = 2), Other FNS Infection (*n* = 4).*

^*f*^With candidiuria: A total of 40 cases. FS Infection (*n* = 20), FNS Infection (*n* = 20); FNS CC2 Infection (*n* = 8), Other FNS Infection (*n* = 12).

*P<0.05, the difference was statistically significant.

## Discussion

In this study, we found 14 new alleles and 27 new DSTs, accounting for 49.1% (27/55) of all DSTs, indicating a high diversity and novelty of *C. tropicalis* isolates in Wuhan. The clinical samples analyzed were from various sources, and no genotype was associated with the infection sites. The local epidemic types (DST506, DST525, and DST546) were all highly resistant to azoles and were found in various samples. This is consistent with the study of Wu et al. (2019), that human anatomical sites do not affect MLST genotypes of *C. tropicalis*. In a study in Taiwan (Li et al., 2009), strains from different anatomical sites of unrelated people shared a common MLST genotype (DST140), which was considered evidence of clonal expansion because the strains were able to grow in high fluconazole concentrations. The prevalence of azole-resistant DSTs in Wuhan can also be considered as the survival of the fittest under high drug concentrations.

Previously, no MLST study of *C. tropicalis* has been conducted in central China. Among the 87 strains, 15 strains (4 DSTs) formed a major FNS cluster, a finding of great value to local medical treatment. When analyzed with the global database and previous studies (Wang Y. et al., 2016; Jin et al., 2018; Chen et al., 2019), these DSTs were found in Beijing, Taiwan, and Shanghai (Supplementary Table S3), and were combined with 13 other DSTs to form a larger cluster (CC2). This phenomenon confirms the gene flow (sharing of multi-loci genotypes across geographic scales) among geographical populations in the global study (Wu et al., 2019). CC2 is a common large cluster of FNS strains in China, without foreign strains. Therefore, it is of great significance to study the drug resistance mechanism and origin of CC2, especially the presumptive founder DST225. Previous studies showed that the *ERG11* mutation, whether or not combined *MDR1* overexpression to produce high-grade fluconazole and other azole-resistant *C. tropicalis* belong to CC2, including DST225 and gene-related DSTs (Chew et al., 2017; Jin et al., 2018). For the source of CC2 strains, in this study, CC2 strains had four possible nosocomial transmissions, two times in the same ward and two times in different wards. The research of Asticcioli et al. (2007) indicates that sharing medical equipment and instruments may be the cause of hospital transmission among different wards. Nosocomial infection may be caused not only by direct contact between patients but also by cross-transmission of medical personnel, devices, instruments, etc. However, these strains were not consistently detected during surveillance. Two environmental surveys have been conducted in China. In one, DST225 strains were isolated from fruits and patients in different hospitals and showed cross-resistance to fluconazole and triadimenol (an azole fungicide) (Lo et al., 2017). The other was an analysis of soil samples. Extensive use of triazole fungicides in agriculture has created azole-resistant strains and passed on to human hosts (Yang et al., 2012). Considering the presence of DST225 and gene-related DSTs strains from patients who had never been exposed to antifungal agents in this study and other two reports in China (Jin et al., 2018; Chen et al., 2019), as well as the high fungicide burden in Asia (Stensvold et al., 2012), we recommend that patients can obtain FNS *C. tropicalis* from the community environment, leading to cross-transmission in the hospital. These findings underscore the importance of active surveillance of FNS *C. tropicalis* in agriculture, hospitals, and the community.

In the global analysis, of the 25 CCs (≥3 DSTs) listed, FNS rates of 8 CCs exceeded 80% and FS rates exceeded 90% in 7 CCs (Supplementary Table S2). This separation of resistant and susceptible strains suggests that they have unique genetic backgrounds. The susceptibility of Asian strains to azoles is lower than that of Europe, America, and Oceania, and FNS CCs have not been seen outside Asia. This is consistent with many previous studies (Corzo-Leon et al., 2014; Huang et al., 2014; Fernández-Ruiz et al., 2015; Tan et al., 2016; Wang H. et al., 2016; Fan et al., 2017; Teo et al., 2017; Wu P. F. et al., 2017; Morris et al., 2018; Scordino et al., 2018; Beyer et al., 2019; Chen et al., 2019). Three FNS CCs from three Asian countries (China: CC2, Singapore: CC4, India: CC17) were independent clusters, but they all appeared after 2011 and may be closely related genetically. CCs from China (CC2, 5–7, 10, 12, 13, 15, 20, 21, 22, 23, and 25), Singapore (CC4) and India (CC17 and CC18) contained almost no strains from other countries. CCs from the United Kingdom (CC8) and Italy (CC23) were all FS isolates. These reflect the differences of epidemic clusters in different geographical locations. Obviously, MLST analysis is of great significance to the epidemiological study of FS and FNS *C. tropicalis* isolates and is helpful to trace their origins. However, our mining of information is limited. More than 70% of the strains in the analysis came from China, whereas studies in Europe and America were less and earlier, and many regions and countries did not have representative strains. This statistical imbalance does not mean that *C. tropicalis* is of low clinical importance in these countries. To some extent, it reflects the efforts of medical mycology researchers from different countries and regions. As a recent study demonstrated, researches on medical fungi have changed dramatically, with a growing number of papers coming from emerging economies such as China, India, and Brazil (Chaturvedi et al., 2018). Our analysis of strains with drug susceptibility information in the database shows that azole-resistance is closely related to genetic background. Research teams in various countries and regions should try to upload the drug susceptibility information of strains when they discover new DSTs, so as to more comprehensively analyze the genetic information and resistance mechanism in different regions, which may also conducive to antifungal drug research.

Multiple studies have shown that patients with azole-resistant *C. tropicalis* candidaemia tend to have higher mortality (Falagas et al., 2010; Giri and Kindo, 2012; Silva et al., 2012; Pahwa et al., 2014). Other studies do not support this view (Brosh-Nissimov and Ben-Ami, 2015; Fernández-Ruiz et al., 2015, 2017; Chen et al., 2019). In our study, there was no statistical significance between patient (with pulmonary candidiasis/with candidemia/with candidiuria) outcomes and azole-resistance and CCs. Among the three factors related to FNS *C. tropicalis* infection, antifungal exposure has been recognized in previous studies (Choi et al., 2016; Chen et al., 2019), but heart disease and hospitalization in the medical wards were unique to our hospital. In Taiwan, moderate to severe liver disease was a unique risk factor for FNS *C. tropicalis* infection (Chen et al., 2019). Hospitalization in medical wards is more likely to lead to FNS *C. tropicalis* infections in our hospital (*P* < 0.05). The FNS rate in medical wards was indeed higher than that in other wards, and most of the six possible nosocomial transmissions were transmitted by FNS *C. tropicalis* in medical wards (Figure 1). These findings suggest that we should further strengthen the monitoring and control of nosocomial infection in the medical wards of the hospital. The downside of this study is that we only conducted it in a single medical center for 12 months. The sample size for risk factor assessment is small, and larger population studies may yield more comprehensive results.

We have submitted new information to the *C. tropicalis* MLST database, and combined with the data from all over the world, elaborated the correlation between FNS/FS strains and the genetic characteristics and geographical locations, and tracked down the FNS clusters in some regions. Since FNS *C. tropicalis* clones may be associated with the use of azole antifungal agents in agriculture and cross-transmission and antifungal exposure in hospitals, further study of strain transmission requires a long period of extensive surveillance from agriculture, hospitals, and the community.

## Data Availability Statement

Publicly available datasets were analyzed in this study. This data can be found here: https://pubmlst.org/ctropicalis/.

## Ethics Statement

The studies involving human participants were reviewed and approved by the Clinical Research Ethics Committee of Renmin Hospital of Wuhan University (WDRY2020-K182). Written informed consent from the participants was not required to participate in this study in accordance with the national legislation and the institutional requirements.

## Author Contributions

QW, KT, and YH collected *C. tropicalis* strains and patient case data. QW and DT completed the antifungal susceptibility test and MLST experiment. QW, DT, and JG analyzed the results. CL guided the whole process. All authors assisted in writing the manuscript.

## Conflict of Interest

The authors declare that the research was conducted in the absence of any commercial or financial relationships that could be construed as a potential conflict of interest.

## Footnotes

1 http://pubmlst.org/ctropicalis/

2 https://www.geneious.com/

3 https://iupac.org/

4 http://pubmlst.org/ctropicalis/

5 http://www.phyloviz.net/

6 http://www.megasoftware.net

7 https://www.applied-maths.com/

8 https://www.ibm.com/analytics/spss-statistics-software

## References

[B1] Arrua J. M. Rodrigues L. A. Pereira F. O. Lima E. O. (2015). Prevalence of *Candida tropicalis* and *Candida krusei* in onychomycosis in João Pessoa, Paraiba, Brazil from 1999 to 2010. *An. Acad. Bras. Cienc.* 87 1819–1822. 10.1590/0001-3765201520130418 26375021

[B2] Asticcioli S. Nucleo E. Perotti G. Matti C. Sacco L. Pagani L. (2007). *Candida albicans* in a neonatal intensive care unit: antifungal susceptibility and genotypic analysis. *N. Microbiol.* 30 303–307. 17802915

[B3] Beyer R. Spettel K. Zeller I. Lass-Flörl C. Achleitner D. Krause R. (2019). Antifungal susceptibility of yeast bloodstream isolates collected during a 10-year period in Austria. *Mycoses* 62 357–367. 10.1111/myc.12892 30636016

[B4] Brosh-Nissimov T. Ben-Ami R. (2015). Differential association of fluconazole dose and dose/MIC ratio with mortality in patients with *Candida albicans* and *non-albicans* bloodstream infection. *Clin. Microbiol. Infect.* 21 1011–1017. 10.1016/j.cmi.2015.07.005 26183300

[B5] Chaturvedi V. Bouchara J. P. Hagen F. Alastruey-Izquierdo A. Badali H. Bocca A. L. (2018). Eighty years of Mycopathologia: a retrospective analysis of progress made in understanding human and animal fungal pathogens. *Mycopathologia* 183 859–877. 10.1007/s11046-018-0306-1 30506286

[B6] Chen P. Y. Chuang Y. C. Wu U. I. Sun H. Y. Wang J. T. Sheng W. H. (2019). Clonality of Fluconazole-Nonsusceptible *Candida tropicalis* in Bloodstream Infections, Taiwan, 2011-2017. *Emerg. Infect. Dis.* 25 1660–1667. 10.3201/eid2509.190520 31441426 PMC6711239

[B7] Chew K. L. Cheng J. W. S. Jureen R. Lin R. T. P. Teo J. W. P. (2017). ERG11 mutations are associated with high-level azole resistance in clinical *Candida tropicalis* isolates, a Singapore study. *Mycoscience* 58 111–115. 10.1016/j.myc.2016.11.001

[B8] Choi M. J. Won E. J. Shin J. H. Kim S. H. Lee W. G. Kim M. N. (2016). Resistance mechanisms and clinical features of fluconazole-nonsusceptible *Candida tropicalis* isolates compared with fluconazole-less-susceptible isolates. *Antimicrob. Agents Chemother.* 60 3653–3661. 10.1128/AAC.02652-15 27044550 PMC4879413

[B9] CLSI (2017a). *Performance Standards for Antifungal Susceptibility Testing of Yeasts. 1st ed. CLSI standard M60.* Wayne, PA: Clinical and Laboratory Standards Institute.

[B10] CLSI (2017b). *Reference Method for Broth Dilution Antifungal Susceptibility Testing of Yeasts. 4th ed. CLSI standard M27.* Wayne, PA: Clinical and Laboratory Standards Institute.

[B11] CLSI (2018). *Epidemiological Cutoff Values for Antifungal Susceptibility Testing. 2nd ed. CLSI standard M59.* Wayne, PA: Clinical and Laboratory Standards Institute.

[B12] Corzo-Leon D. E. Alvarado-Matute T. Colombo A. L. Cornejo-Juarez P. Cortes J. Echevarria J. I. (2014). Surveillance of *Candida* spp bloodstream infections: epidemiological trends and risk factors of death in two Mexican tertiary care hospitals. *PLoS One* 9:e97325. 10.1371/journal.pone.0097325 24830654 PMC4022628

[B13] Diba K. Makhdoomi K. Nasri E. Vaezi A. Javidnia J. Gharabagh D. J. (2018). Emerging *Candida* species isolated from renal transplant recipients: species distribution and susceptibility profiles. *Microb. Pathog.* 125 240–245. 10.1016/j.micpath.2018.09.026 30240817

[B14] Dong D. Li Z. Zhang L. Jiang C. Mao E. Wang X. (2015). Clinical and microbiological investigation of fungemia from four hospitals in China. *Mycopathologia* 179 407–414. 10.1007/s11046-014-9855-0 25720562

[B15] Falagas M. E. Roussos N. Vardakas K. Z. (2010). Relative frequency of *albicans* and the various *non-albicans Candida* spp among candidemia isolates from inpatients in various parts of the world: a systematic review. *Int. J. Infect. Dis.* 14 e954–e966. 10.1016/j.ijid.2010.04.006 20797887

[B16] Fan X. Xiao M. Liao K. Kudinha T. Wang H. Zhang L. (2017). Notable increasing trend in azole non-susceptible *Candida tropicalis* causing invasive candidiasis in China (August 2009 to July 2014): molecular epidemiology and clinical azole consumption. *Front. Microbiol.* 8:464. 10.3389/fmicb.2017.00464 28382028 PMC5360734

[B17] Fernández-Ruiz M. Guinea J. Lora-Pablos D. Zaragoza Ó. Puig-Asensio M. Almirante B. (2017). Impact of fluconazole susceptibility on the outcome of patients with candidaemia: data from a population-based surveillance. *Clin. Microbiol. Infect.* 23 672.e1–672.e11. 10.1016/j.cmi.2017.01.014 28143788

[B18] Fernández-Ruiz M. Puig-Asensio M. Guinea J. Almirante B. Padilla B. Almela M. (2015). *Candida tropicalis* bloodstream infection: Incidence, risk factors and outcome in a population-based surveillance. *J. Infect.* 71 385–394. 10.1016/j.jinf.2015.05.009 26033696

[B19] Fesharaki S. H. Haghani I. Mousavi B. Kargar M. L. Boroumand M. Anvari M. S. (2013). Endocarditis due to a co-infection of *Candida albicans* and *Candida tropicalis* in a drug abuser. *J. Med. Microbiol.* 62 1763–1767. 10.1099/jmm.0.060954-0 23973985

[B20] Giri S. Kindo A. J. (2012). A review of *Candida* species causing blood stream infection. *Ind. J. Med. Microbiol.* 30 270–278. 10.4103/0255-0857.99484 22885191

[B21] Hillis D. M. Bull J. J. (1993). An empirical test of bootstrapping as a method for assessing confidence in phylogenetic analyses. *Syst. Biol.* 42 182–192. 10.1093/sysbio/42.2.182

[B22] Huang Y. T. Liu C. Y. Liao C. H. Chung K. P. Sheng W. H. Hsueh P. R. (2014). Antifungal susceptibilities of *Candida* isolates causing bloodstream infections at a medical center in Taiwan, 2009-2010. *Antimicrob. Agents Chemother.* 58 3814–3819. 10.1128/AAC.01035-13 24752274 PMC4068559

[B23] Jin L. Cao Z. Wang Q. Wang Y. Wang X. Chen H. (2018). *MDR1* overexpression combined with *ERG11* mutations induce high-level fluconazole resistance in *Candida tropicalis* clinical isolates. *BMC Infect. Dis.* 18:162. 10.1186/s12879-018-3082-0 29631565 PMC5891969

[B24] Kumar S. Kalam K. Ali S. Siddiqi S. Baqi S. (2014). Frequency, clinical presentation and microbiological spectrum of candidemia in a tertiary care center in Karachi, Pakistan. *J. Pak. Med. Assoc.* 64 281–285. 24864600

[B25] Lee H. S. Shin J. H. Choi M. J. Won E. J. Kee S. J. Kim S. H. (2017). Comparison of the Bruker Biotyper and VITEK MS matrix-assisted laser desorption/ionization time-of-flight mass spectrometry systems using a formic acid extraction method to identify common and uncommon yeast isolates. *Ann. Lab. Med.* 37 223–230. 10.3343/alm.2017.37.3.223 28224768 PMC5339094

[B26] Li S. Y. Yang Y. L. Lin Y. H. Ko H. C. Wang A. H. Chen K. W. (2009). Two closely related Fluconazole-resistant *Candida tropicalis* clones circulating in Taiwan from 1999 to 2006. *Microb. Drug. Resist.* 15 205–210. 10.1089/mdr.2009.0915 19728779

[B27] Lo H. J. Tsai S. H. Chu W. L. Chen Y. Z. Zhou Z. L. Chen H. F. (2017). Fruits as the vehicle of drug resistant pathogenic yeasts. *J. Infect.* 75 254–262. 10.1016/j.jinf.2017.06.005 28648496

[B28] Magri M. M. Gomes-Gouvêa M. S. de Freitas V. L. Motta A. L. Moretti M. L. Shikanai-Yasuda M. A. (2013). Multilocus sequence typing of *Candida tropicalis* shows the presence of different clonal clusters and fluconazole susceptibility profiles in sequential isolates from candidemia patients in Sao Paulo, Brazil. *J. Clin. Microbiol*. 51, 268–277. 10.1128/JCM.02366-121 23152555 PMC3536249

[B29] Mizusawa M. Miller H. Green R. Lee R. Durante M. Perkins R. (2017). Can multidrug-resistant *Candida auris* be reliably identified in clinical microbiology laboratories? *J. Clin. Microbiol.* 55 638–640. 10.1128/JCM.02202-16 27881617 PMC5277535

[B30] Morris A. J. Rogers K. McKinney W. P. Roberts SA. Freeman J. T. (2018). Antifungal susceptibility testing results of New Zealand yeast isolates, 2001-2015: Impact of recent CLSI breakpoints and epidemiological cut-off values for *Candida* and other yeast species. *J. Glob. Antimicrob. Resist.* 14 72–77. 10.1016/j.jgar.2018.02.014 29486358

[B31] Pahwa N. Kumar R. Nirkhiwale S. Bandi A. (2014). Species distribution and drug susceptibility of *Candida* in clinical isolates from a tertiary care centre at Indore. *Ind. J. Med. Microbiol.* 32 44–48. 10.4103/0255-0857.124300 24399387

[B32] Scordino F. Giuffrè L. Barberi G. Marino Merlo F. Orlando M. G. Giosa D. (2018). Multilocus Sequence Typing Reveals a New Cluster of Closely Related *Candida tropicalis* Genotypes in Italian Patients With Neurological Disorders. *Front Microbiol*. 9:679. 10.3389/fmicb.2018.00679 29696003 PMC5904457

[B33] Silva S. Negri M. Henriques M. Oliveira R. Williams D. W. Azeredo J. (2012). *Candida g*labrata, Candida parapsilosis and Candida tropicalis: biology, epidemiology, pathogenicity and antifungal resistance. *FEMS Microbiol. Rev.* 36 288–305. 10.1111/j.1574-6976.2011.00278 21569057

[B34] Stensvold C. R. Jørgensen L. N. Arendrup M. C. (2012). Azole-resistant invasive aspergillosis: relationship to agriculture. *Curr. Fungal Infect. Rep.* 6 178–191. 10.1007/s12281-012-0097-7

[B35] Tan B. H. Chakrabarti A. Li R. Y. Patel A. K. Watcharananan S. P. Liu Z. (2015). Incidence and species distribution of candidaemia in Asia: a laboratory-based surveillance study. *Clin. Microbiol. Infect.* 21 946–953. 10.1016/j.cmi.2015.06.010 26100373

[B36] Tan T. Y. Hsu L. Y. Alejandria M. M. Chaiwarith R. Chinniah T. Chayakulkeeree M. (2016). Antifungal susceptibility of invasive *Candida* bloodstream isolates from the Asia-Pacific region. *Med. Mycol.* 54 471–477. 10.1093/mmy/myv114 26868904

[B37] Tavanti A. Davidson A. D. Johnson E. M. Maiden M. C. Shaw D. J. Gow N. A. (2005). Multilocus sequence typing for differentiation of strains of *Candida tropicalis*. *J. Clin. Microbiol.* 43 5593–5600. 10.1128/JCM.43.11.5593-5600.2005 16272492 PMC1287820

[B38] Teo J. Q. Candra S. R. Lee S. J. Chia S. Y. Leck H. Tan A. L. (2017). Candidemia in a major regional tertiary referral hospital-epidemiology, practice patterns and outcomes. *Antimicrob. Resist. Infect. Control* 6:27. 10.1186/s13756-017-0184-1 28293420 PMC5346229

[B39] Wang H. Xu Y. C. Hsueh P. R. (2016). Epidemiology of candidemia and antifungal susceptibility in invasive *Candida* species in the Asia-Pacific region. *Future Microbiol.* 11 1461–1477. 10.2217/fmb-2016-0099 27750452

[B40] Wang H. C. Huang J. C. Lin Y. H. Chen Y. H. Hsieh M. I. Choi P. C. (2018). Prevalence, mechanisms and genetic relatedness of the human pathogenic fungus Aspergillus fumigatus exhibiting resistance to medical azoles in the environment of Taiwan. *Environ. Microbiol.* 20 270–280. 10.1111/1462-2920.13988 29124846

[B41] Wang Y. Shi C. Liu J. Y. Li W. J. Zhao Y. Xiang M. J. (2016). Multilocus sequence typing of *Candida tropicalis* shows clonal cluster enrichment in azole-resistant isolates from patients in Shanghai, China. *Infect. Genet. Evol.* 44 418–424. 10.1016/j.meegid.2016.07.026 27456280

[B42] Wu J. Y. Guo H. Wang H. M. Yi G. H. Zhou L. M. He X. W. (2017). Multilocus sequence analyses reveal extensive diversity and multiple origins of fluconazole resistance in *Candida tropicalis* from tropical China. *Sci. Rep.* 7:42537. 10.1038/srep42537 28186162 PMC5301247

[B43] Wu J. Y. Zhou D. Y. Zhang Y. Mi F. Xu J. (2019). Analyses of the Global Multilocus Genotypes of the Human Pathogenic Yeast *Candida tropicalis*. *Front. Microbiol.* 10:900. 10.3389/fmicb.2019.00900 31080446 PMC6497803

[B44] Wu P. F. Liu W. L. Hsieh M. H. Hii I. M. Lee Y. L. Lin Y. T. (2017). Epidemiology and antifungal susceptibility of candidemia isolates of *non-albicans Candida* species from cancer patients. *Emerg. Microbes Infect.* 6:e87. 10.1038/emi.2017.74 29018251 PMC5658770

[B45] Yang Y. L. Lin C. C. Chang T. P. Lauderdale T. L. Chen H. T. Lee C. F. (2012). Comparison of human and soil *Candida tropicalis* isolates with reduced susceptibility to fluconazole. *PLoS One* 7:e34609. 10.1371/journal.pone.0034609 22496832 PMC3320620

[B46] Zuza-Alves D. L. Silva-Rocha W. P. Chaves G. M. (2017). An update on *Candida tropicalis* based on basic and clinical approaches. *Front. Microbiol.* 8:1927. 10.3389/fmicb.2017.01927 29081766 PMC5645804

