# Supplementary material for: Multilocus Sequence Typing Reveals Clonality of Fluconazole-Nonsusceptible Candida tropicalis: A Study From Wuhan to the Global
Source: Front Microbiol. 2020 Nov 17;11:554249. doi: 10.3389/fmicb.2020.554249 (PMC7705220; doi:10.3389/fmicb.2020.554249)
Supplement: Supplementary file 1 [file Table_1.PDF]

Supplementary Table S1 Clinical information, antifungal susceptibility, and MLST analyses of 87 *Candida tropicalis* isolates from Wuhan

Isolate	Patient	Collection Date	Source	Ward	Outcome	MIC (μg/mL)			DST	Clonal complex
						FLC	VRC	ITC		
CTR-1	**P1**	07/12/2018	Urine	Geriatric	Survival	>64	2	1	506	2
CTR-2	P2	07/12/2018	Catheter tip	Pancreatic	Survival	>64	2	1	506	2
CTR-3	P3	10/12/2018	Urine	Pancreatic	Survival	>64	16	1	506	2
CTR-4	P4	22/12/2018	Blood	ICU	Death	64	>16	16	525	6
CTR-5	P5	20/01/2019	Blood	RICU	Death	4	<0.031	0.25	980*	Singleton
CTR-6	P6	26/01/2019	Urine	Neurosurgery	Survival	32	16	4	615	Singleton
CTR-7	**P7**	04/02/2019	Urine	Geriatric	Death	>64	8	16	525	6
CTR-8	P8	12/02/2019	Urine	RICU	Survival	2	0.25	0.25	987*	Singleton
CTR-9	P9	18/02/2019	Blood	ICU	Death	0.25	0.0625	0.125	992*	Singleton
CTR-10	P10	05/03/2019	Vaginal swab	Gynecology	Survival	64	16	16	982*	Singleton
CTR-11	P11	07/03/2019	Urine	Geriatric	Survival	0.5	0.125	0.125	330	Singleton
CTR-12	P12	09/03/2019	Urine	Neurosurgery	Survival	2	0.125	0.25	437	Singleton
CTR-13	P13	23/03/2019	Vaginal swab	Reproductive	Survival	0.5	0.0313	0.125	994*	Singleton
CTR-14	P14	24/03/2019	Vaginal swab	Gynecology	Survival	1	0.0625	0.25	343	3
CTR-15	P15	27/03/2019	Bile	Hepatobiliary	Survival	1	0.125	0.12	337	7
CTR-16	P16	27/03/2019	Urine	Urology	Survival	0.5	0.0625	0.125	169	Singleton
CTR-17	P17	31/03/2019	Urine	Urology	Survival	0.5	0.0625	0.25	346	8
CTR-18	P18	02/04/2019	Vaginal swab	Gynecology	Survival	32	16	16	615	Singleton
CTR-19	P19	06/04/2019	Urine	ICU	Death	0.5	0.0313	0.125	730	5
CTR-20	P20	07/04/2019	Urine	ICU	Survival	32	4	8	615	Singleton
CTR-21	P21	14/04/2019	Vaginal swab	Gynecology	Survival	0.125	0.0313	0.25	169	Singleton
CTR-22	P22	20/04/2019	Catheter tip	Cardiovascular	Survival	1	0.0313	0.25	993*	Singleton
CTR-23	P23	21/04/2019	Urine	RICU	Death	64	16	16	482	Singleton
CTR-24	P24	26/04/2019	Urine	Neurology	Survival	64	16	2	331	1
CTR-25	P25	27/04/2019	Vaginal swab	Gynecology	Survival	32	>16	>16	525	6
CTR-26	P26	27/04/2019	Urine	Cardiovascular	Survival	8	16	8	331	1
CTR-27	P27	16/05/2019	Vaginal swab	Reproductive	Survival	8	16	>16	983*	Singleton
CTR-28	P28	20/05/2019	Vaginal swab	Reproductive	Survival	0.25	0.0313	0.25	984*	Singleton
CTR-29	P29	24/05/2019	Bile	Hepatobiliary	Survival	64	1	0.5	546	2
CTR-30	P30	27/05/2019	Vaginal swab	Gynecology	Survival	0.125	0.0313	0.25	723	3
CTR-31	P31	30/05/2019	Vaginal swab	Reproductive	Survival	0.125	0.0313	0.25	995*	Singleton
CTR-32	P32	04/06/2019	Vaginal swab	Gynecology	Survival	1	<0.031	0.25	901	Singleton
CTR-33	P33	05/06/2019	Sputum	RICU	Death	64	8	8	506	2
CTR-34	P34	05/06/2019	Vaginal swab	Gynecology	Survival	32	16	16	996*	Singleton
CTR-35	P35	11/06/2019	Urine	Neurology	Survival	0.5	0.0625	0.125	184	4
CTR-36	P36	12/06/2019	Sputum	Geriatric	Survival	0.5	<0.031	0.0625	833	4
CTR-37	**P7**	12/06/2019	Urine	Geriatric	Death	64	>16	>16	525	6
CTR-38	P37	18/06/2019	Sputum	Cardiovascular	Survival	0.25	0.0313	0.125	394	1
CTR-39	P38	25/06/2019	Ascites	Pancreatic	Survival	1	0.0313	0.25	346	8
CTR-40	P39	30/06/2019	Bile	Interventional	Survival	32	8	>16	997*	Singleton
CTR-41	P40	09/07/2019	Blood	Pancreatic	Survival	2	0.25	0.5	522	7
CTR-42	P41	28/07/2019	Blood	Hematology	Survival	0.25	0.0625	0.125	991*	Singleton
CTR-43	P42	03/08/2019	Vaginal swab	Gynecology	Survival	32	4	1	225	2

CTR-44	P43	07/08/2019	Vaginal swab	Reproductive	Survival	0.25	0.125	0.5	998*	3
CTR-45	P44	11/08/2019	Blood	Pancreatic	Survival	2	0.25	0.5	981*	1
CTR-46	P45	15/08/2019	Urine	Oncology	Survival	>64	8	1	225	2
CTR-47	P46	21/08/2019	Urine	Urology	Survival	16	8	8	525	6
CTR-48	P47	21/08/2019	Blood	ICU	Survival	2	0.25	0.25	522	7
CTR-49	P48	23/08/2019	Vaginal swab	Reproductive	Survival	0.25	0.0625	0.25	331	1
CTR-50	P49	23/08/2019	Urine	Nephrology	Survival	>64	2	4	376	2
CTR-51	P50	24/08/2019	Vaginal swab	Reproductive	Survival	0.5	0.25	1	852	Singleton
CTR-52	P51	24/08/2019	Vaginal swab	Reproductive	Survival	0.25	0.0313	0.5	139	4
CTR-53	P52	29/08/2019	Urine	Urology	Survival	1	1	2	999*	Singleton
CTR-54	P53	06/09/2019	Blood	Transplantation	Survival	4	0.25	0.5	437	Singleton
CTR-55	P54	11/09/2019	Vaginal swab	Gynecology	Survival	0.125	0.125	0.25	1002*	9
CTR-56	P55	11/09/2019	Vaginal swab	Reproductive	Survival	0.5	0.25	2	988*	Singleton
CTR-57	P56	13/09/2019	Urine	Gynecology	Survival	16	16	4	976*	Singleton
CTR-58	P57	18/09/2019	Bile	Hepatobiliary	Survival	0.5	0.25	0.5	434	1
CTR-59	P58	28/09/2019	Vaginal swab	Reproductive	Survival	0.5	0.0313	0.5	985*	8
CTR-60	P59	30/09/2019	Urine	Urology	Survival	0.25	0.125	0.25	403	Singleton
CTR-61	P60	30/09/2019	Urine	Neurology	Death	64	8	1	546	2
CTR-62	P61	01/10/2019	Urine	ICU	Death	0.25	0.0625	0.25	346	8
CTR-63	P62	03/10/2019	Urine	Neurology	Survival	64	2	1	546	2
CTR-64	P63	03/10/2019	Urine	Oncology	Survival	64	2	2	546	2
CTR-65	P64	04/10/2019	Urine	Urology	Survival	1	0.25	0.25	989*	Singleton
CTR-66	P65	05/10/2019	Vaginal swab	Reproductive	Survival	0.25	0.0313	0.5	139	4
CTR-67	P66	07/10/2019	Urine	RICU	Death	0.25	0.0313	0.25	978*	6
CTR-68	P67	08/10/2019	Urine	Urology	Survival	32	4	2	990*	Singleton
CTR-69	**P68**	09/10/2019	Urine	ICU	Death	>64	4	4	506	2
CTR-70	P69	11/10/2019	Urine	Endocrinology	Survival	0.5	0.25	0.5	434	1
CTR-71	P70	14/10/2019	Urine	Urology	Survival	0.25	0.0313	0.125	394	1
CTR-72	P71	16/10/2019	Vaginal swab	Gynecology	Survival	0.25	0.0313	0.5	1000*	Singleton
CTR-73	**P68**	20/10/2019	Blood	ICU	Death	>64	4	4	506	2
CTR-74	**P1**	23/10/2019	Urine	Geriatric	Survival	64	2	1	506	2
CTR-75	P72	23/10/2019	Urine	ICU	Death	0.25	0.125	0.125	977*	5
CTR-76	P73	29/10/2019	Urine	Geriatric	Death	32	16	0.5	979*	Singleton
CTR-77	P74	30/10/2019	Urine	Urology	Survival	1	0.0625	0.125	184	4
CTR-78	P75	31/10/2019	Urine	Transplantation	Survival	>64	8	1	546	2
CTR-79	P76	01/11/2019	Urine	Urology	Survival	0.5	0.25	0.5	489	9
CTR-80	P77	01/11/2019	Urine	ICU	Survival	0.25	0.0625	0.25	343	3
CTR-81	P78	05/11/2019	Urine	Urology	Survival	0.25	0.0625	0.25	346	8
CTR-82	P79	07/11/2019	Urine	Urology	Survival	0.5	<0.031	0.25	978*	6
CTR-83	P80	11/11/2019	Urine	RICU	Death	64	8	4	923	Singleton
CTR-84	P81	18/11/2019	Sputum	Oncology	Death	0.25	0.0313	0.5	184	4
CTR-85	P82	23/11/2019	Blood	Pancreatic	Survival	0.5	0.0625	0.125	1001*	Singleton
CTR-86	P83	26/11/2019	Blood	Oncology	Survival	8	0.25	0.5	986*	Singleton
CTR-87	P84	30/11/2019	Urine	Oncology	Death	64	16	16	181	Singleton

Isolates from the same patient indicated by bold type.

* *New DSTs in C. tropicalis MLST database (http://pubmlst.org/ctropicalis/).*

Resistant or Non-wildtype   *Intermediate*   *Susceptible or Wildtype*
