# Supplementary material for: Multilocus Sequence Typing Reveals Clonality of Fluconazole-Nonsusceptible Candida tropicalis: A Study From Wuhan to the Global
Source: Front Microbiol. 2020 Nov 17;11:554249. doi: 10.3389/fmicb.2020.554249 (PMC7705220; doi:10.3389/fmicb.2020.554249)
Supplement: Supplementary file 2 [file Table_2.PDF]

Supplementary Table S2 Detailed information of *Candida tropicalis* isolates in CC1~CC25 obtained by goeBURST analysis

Clonal complex	DST	Isolate	Anatomical source	Year	Country (region)	FLC MIC (μg/mL)	S / I / R	FNS Rate
CC1	45	AM2003/0078	blood	2003	UK	0.25	S	80.8% (42/52)
		b30980/5/04	blood	2004		2	S	
		YM060100	sputum/BAL	2006	Taiwan, China	>64	R	
	98	YM990131	urine	1999	Taiwan, China	>64	R	
		YM990135	sputum/BAL			>64	R	
		YM060450	sputum/BAL	2006		>64	R	
		YM060451	sputum/BAL			>64	R	
		YM060647	sputum/BAL			>64	R	
		YM060529	sputum/BAL			>64	R	
		YM060547	blood			>64	R	
	137	YM990148	urine	1999	Taiwan, China	0.5	S	
	140	YM990490	urine	1999	Taiwan, China	>64	R	
		YM990603	sputum/BAL			>64	R	
		YM990592	blood			>64	R	
		YM020919	sputum/BAL			1	S	
		YM990645	urine			>64	R	
		YM990647	urine			0.13	S	
		YM990649	urine			>64	R	
		YM990659	blood			>64	R	
		YM990660	urine			>64	R	
		YM990662	urine			>64	R	
		YM990275	urine			>64	R	
		YM990577	sputum/BAL			>64	R	
		YM990537	urine			>64	R	
		YM020309	urine	2002		4	I	
		YM020273	sputum/BAL			4	I	
		YM060327	urine	2006		>64	R	
		YM060509	sputum/BAL			>64	R	
		YM060098	sputum/BAL			>64	R	
		YM060102	sputum/BAL			>64	R	
		YM060173	urine			>64	R	
		YM060828	blood			>64	R	
		NHUE10	—			>64	R	
		NHUE56	—			>64	R	
		NHUE48	—	2008		>64	R	
	144	YM990458	blood	1999	Taiwan, China	>64	R	
		YM020294	urine	2002		16	R	
	147	YM990554	urine	1999	Taiwan, China	>64	R	
	168	YM060136	blood	2006	Taiwan, China	0.5	S	
		NHUE23	—	2007		>64	R	
	179	YM060776	blood	2006	Taiwan, China	>64	R	
		YM060175	urine			>64	R	
	180	YM060144	urine	2006	Taiwan, China	>64	R	

	**181**	YM060590	urine	2006	Taiwan, China	>64	**R**	
		CTR-87	Urine	2019	Wuhan, China	64	**R**	
	**182**	YM060805	urine	2006	Taiwan, China	>64	**R**	
	**229**	NHUE27	—	2007	Taiwan, China	1	**S**	
	**572**	YM140212	sputum/BAL	2014	Taiwan, China	4	**I**	
	**820**	F2017a060	blood	2017	Taiwan, China	2	**S**	
	**829**	F2017c051	blood	2017	Taiwan, China	2	**S**	
	**830**	F2017c080	blood	2017	Taiwan, China	2	**S**	
	**911**	YM140896	blood	2014	Taiwan, China	64	**R**	
**CC2**	**225**	CTR-43	Vaginal swab	2019	Wuhan, China	32	**R**	**100%** (42/42)
		CTR-46	Urine			>64	**R**	
	**376**	CTR-50	Urine	2019	Wuhan, China	>64	**R**	
	**505**	572	sputum/BAL	2012	Shanghai, China	64	**R**	
	**506**	573	sputum/BAL	2013	Shanghai, China	64	**R**	
		YM140066	—	2014	Taiwan, China	64	**R**	
		CTR-1	Urine	2018	Wuhan, China	>64	**R**	
		CTR-2	Catheter tip			>64	**R**	
		CTR-3	Urine			>64	**R**	
		CTR-33	sputum/BAL	2019		64	**R**	
		CTR-69	Urine			>64	**R**	
		CTR-73	Blood			>64	**R**	
		CTR-74	Urine			64	**R**	
	**507**	574	urine	2013	Shanghai, China	128	**R**	
		575	sputum/BAL			128	**R**	
		576	sputum/BAL			64	**R**	
		577	sputum/BAL			64	**R**	
		578	sputum/BAL			64	**R**	
		579	other superficial source			64	**R**	
		580	blood	2014		64	**R**	
		581	sputum/BAL			64	**R**	
		582	urine			32	**R**	
		583	sputum/BAL			32	**R**	
		584	urine			128	**R**	
		585	other sterile site	2015		32	**R**	
		586	urine			64	**R**	
	**546**	CTR-29	Bile	2019	Wuhan, China	64	**R**	
		CTR-61	Urine			64	**R**	
		CTR-63	Urine			64	**R**	
		CTR-64	Urine			64	**R**	
		CTR-78	Urine			>64	**R**	
	**592**	YFA123445	—	2012	Taiwan, China	64	**R**	
	**593**	YM140586	blood	2014	Taiwan, China	64	**R**	
	**594**	YM140285	urine	2014	Taiwan, China	64	**R**	
	**595**	YM140907	blood	2014	Taiwan, China	4	**I**	
	**596**	YFA121135	—	2012	Taiwan, China	4	**I**	
	**600**	YM140789	urine	2014	Taiwan, China	4	**I**	
	**838**	F2017f039	blood	2017	Taiwan, China	256	**R**	
	**849**	CT105	—	2013	Taiwan, China	64	**R**	
	**855**	CT261	—	2012	Taiwan, China	128	**R**	

	**879**	YFA180849	—	—	Taiwan, China	64	**R**	
	**924**	YM140982	—	2014	Taiwan, China	4	**I**	
**CC3**	**7**	J980160	sputum/BAL	1998	California, USA	2	**S**	34.6% (9/26)
		J980156	blood			0.5	**S**	
	**14**	A427748	blood	1999	Aberdeen, UK	16	**R**	
	**27**	YM060481	urine	2006	Taiwan, China	>64	**R**	
		NCCLS71	—	—	USA	1	**S**	
	**30**	J980157	other sterile site	1998	USA	2	**S**	
	**37**	J980162	sputum/BAL	1998	California, USA	2	**S**	
	**64**	b30488/7/04	sputum/BAL	2004	UK	0.25	**S**	
	**90**	YM990598	sputum/BAL	1999	Taiwan, China	>64	**R**	
		YM020311	urine	2002		16	**R**	
		YM020693	blood			0.5	**S**	
	**134**	YM990138	sputum/BAL	1999	Taiwan, China	0.5	**S**	
		YM020743	blood	2002		0.13	**S**	
		YM060507	sputum/BAL	2006		>64	**R**	
		YM060299	blood			>64	**R**	
		YM060508	sputum/BAL			1	**S**	
		YM060512	sputum/BAL			0.5	**S**	
	**145**	YM990533	sputum/BAL	1999	Taiwan, China	0.5	**S**	
	**153**	YM020274	sputum/BAL	2002	Taiwan, China	0.25	**S**	
	**155**	YM020291	sputum/BAL	2002	Taiwan, China	0.25	**S**	
	**200**	YM060379	blood	2006	Taiwan, China	0.5	**S**	
		YM060800	urine			0.25	**S**	
	**589**	YM140717	urine	2014	Taiwan, China	4	**I**	
	**778**	CT183	—	2012	Taiwan, China	4	**I**	
	**779**	CT205	—	2013	Taiwan, China	0.25	**S**	
	**780**	CT262	—	2012	Taiwan, China	4	**I**	
**CC4**	**499**	K2	blood	2015	Singapore	>256	**R**	**92.9%** (13/14)
		624	blood			>256	**R**	
	**536**	623	blood	2015	Singapore	>256	**R**	
	**537**	625	blood	2015	Singapore	64	**R**	
	**538**	626	other sterile site	2015	Singapore	>256	**R**	
	**539**	627	blood	2015	Singapore	256	**R**	
	**540**	628	blood	2015	Singapore	>256	**R**	
	**541**	629	blood	2015	Singapore	96	**R**	
	**542**	630	blood	2015	Singapore	>256	**R**	
	**543**	631	blood	2015	Singapore	>256	**R**	
	**544**	632	blood	2015	Singapore	>256	**R**	
	**545**	633	blood	2015	Singapore	0.25	**S**	
	**605**	9238	blood	2016	Nanchang, China	4	**I**	
	**608**	12070	blood	2016	Nanchang, China	64	**R**	
**CC5**	**138**	YM990236	urine	1999	Taiwan, China	>64	**R**	21.1% (4/19)
	**139**	YM990268	urine	1999	Taiwan, China	0.5	**S**	
		YM060369	blood	2006		>64	**R**	
		NHUE42	—	2007		0.5	**S**	
		CTR-52	Vaginal swab	2019	Wuhan, China	0.25	**S**	
		CTR-66	Vaginal swab			0.25	**S**	
	**184**	YM060210	urine	2006	Taiwan, China	>64	**R**	

		CTR-35	Urine	2019	Wuhan, China	0.5	S	
		CTR-77	Urine			1	S	
		CTR-84	sputum/BAL			0.25	S	
	**823**	F2017b014	blood	2017	Taiwan, China	1	S	
		F2017b034	blood			1	S	
	**825**	F2017b038	blood	2017	Taiwan, China	2	S	
	**832**	F2017d049	blood	2017	Taiwan, China	1	S	
	**833**	F2017d081	blood	2017	Taiwan, China	1	S	
		F2017f050	blood			1	S	
		CTR-36	sputum/BAL	2019	Wuhan, China	0.5	S	
	**834**	F2017e045	blood	2017	Taiwan, China	1	S	
	**853**	CT195	—	2012	Taiwan, China	32	**R**	
**CC6**	**579**	YM140516	urine	2014	Taiwan, China	4	**I**	55.6% (5/9)
	**580**	YFA120855	—	2012	Wuhan, China	2	S	
	**581**	YFA120853	—	2012	Taiwan, China	2	S	
	**582**	YM140682	sputum/BAL	2014	Taiwan, China	4	**I**	
	**585**	YM140298	urine	2014	Wuhan, China	8	**R**	
	**586**	YFA120274	—	2012	Taiwan, China	2	S	
	**587**	YFA120766	—	2012	Taiwan, China	2	S	
	**848**	CT98	—	2013	Taiwan, China	8	**R**	
	**996**	CTR-34	Vaginal swab	2019	Wuhan, China	32	**R**	
**CC7**	**149**	YM990579	urine	1999	Taiwan, China	>64	**R**	70% (14/20)
		YM060177	urine	2006		>64	**R**	
		YM060097	sputum/BAL			>64	**R**	
		NHUE17	—	2007		>64	**R**	
		NHUE18	—			>64	**R**	
		NHUE19	—			>64	**R**	
		NHUE28	—			>64	**R**	
		NHUE29	—			>64	**R**	
		NHUE30	—			>64	**R**	
		NHUE33	—			>64	**R**	
		NHUE34	—			>64	**R**	
		NHUE40	—			>64	**R**	
	**337**	CTR-15	Bile	2019	Wuhan, China	1	S	
	**522**	607	urine	2013	Shanghai, China	1	S	
		606	blood	2015		0.5	S	
		CTR-41	Blood	2019	Wuhan, China	2	S	
		CTR-48	Blood			2	S	
	**576**	YFA122284	—	2012	Taiwan, China	>64	**R**	
	**577**	YFA121513	—	2012	Taiwan, China	>64	**R**	
	**835**	F2017e077	blood	2017	Taiwan, China	2	S	
**CC8**	**5**	L601	other superficial source	1986	London, UK	2	S	**8.3%** (1/12)
	**13**	L711	sputum/BAL	1986	London, UK	2	S	
		J942213	—	1994	Belgium	4	**I**	
	**18**	L590	vagina	1985	London, UK	1	S	
		L831	sputum/BAL	1986		1	S	
		L501	faeces			0.5	S	
		L474	vagina			1	S	
	**31**	75/035	sputum/BAL	1975	Leeds, UK	1	S	

		A700246	sputum/BAL	1999	Aberdeen, UK	0.13	S	
		b30343/7/04	blood	2004	UK	0.25	S	
		b31429/7/04	blood			0.5	S	
	**65**	b30604/7/04	sputum/BAL	2004	UK	0.25	S	
**CC9**	**158**	YM020671	blood	2002	Taiwan, China	8	R	28.6% (2/7)
	**359**	IRCCS 46	blood	2017	Sicily, Italy	<=1	S	
		IRCCS 47	—			<=1	S	
	**523**	608	sputum/BAL	2012	Shanghai, China	1	S	
	**750**	IRCCS 14	blood	2016	Sicily, Italy	<=1	S	
	**923**	YM140438	urine	2014	Taiwan, China	0.25	S	
	**923**	CTR-83	Urine	2019	Wuhan, China	64	R	
**CC10**	**346**	CTR-17	Urine	2019	Wuhan, China	0.5	S	**0** (0/9)
		CTR-39	Ascites			1	S	
		CTR-62	Urine			0.25	S	
		CTR-81	Urine			0.25	S	
	**503**	569	urine	2012	Shanghai, China	1	S	
	**504**	570	urine	2014	Shanghai, China	0.5	S	
		571	urine			0.5	S	
	**519**	600	sputum/BAL	2014	Shanghai, China	1	S	
	**985**	CTR-59	Vaginal swab	2019	Wuhan, China	0.5	S	
**CC11**	**186**	YM060330	sputum/BAL	2006	Taiwan, China	>64	R	**85.7%** (6/7)
	**187**	YM060371	blood	2006	Taiwan, China	>64	R	
		NHUE39	—	2007		>64	R	
	**188**	YM060088	sputum/BAL	2006	Taiwan, China	>64	R	
		YM060146	other sterile site			>64	R	
	**975**	SP4694	other sterile site	2018	Ontario, Canada	1	S	
		SP4785	other sterile site			32	R	
**CC12**	**331**	CTR-24	Urine	2019	Wuhan, China	64	R	25% (2/8)
		CTR-26	Urine			8	R	
		CTR-49	Vaginal swab			0.25	S	
	**394**	CTR-38	sputum/BAL	2019	Wuhan, China	0.25	S	
		CTR-71	Urine			0.25	S	
	**434**	CTR-58	Bile	2019	Wuhan, China	0.5	S	
		CTR-70	Urine			0.5	S	
	**981**	CTR-45	Blood	2019	Wuhan, China	2	S	
**CC13**	**516**	597	sputum/BAL	2013	Shanghai, China	0.5	S	45.5% (5/11)
	**525**	610	faeces	2012	Shanghai, China	1	S	
		611	urine	2014		0.5	S	
		CTR-4	Blood	2018	Wuhan, China	64	R	
		CTR-7	Urine	2019		>64	R	
		CTR-25	Vaginal swab			32	R	
		CTR-37	Urine			64	R	
		CTR-47	Urine			16	R	
	**526**	612	faeces	2013	Shanghai, China	0.5	S	
	**978**	CTR-67	Urine	2019	Wuhan, China	0.25	S	
		CTR-82	Urine	2019		0.5	S	
**CC14**	**535**	622	blood	2015	Singapore	>256	R	**87.5%** (7/8)
	**606**	10307	blood	2016	Nanchang, China	8	R	
		10285	blood			4	I	

		10471	blood			4	I	
	609	10787-2	blood	2016	Nanchang, China	8	R	
		12127	blood			8	R	
		10787-1	blood			8	R	
	610	10215	blood	2016	Nanchang, China	2	S	
CC15	150	YM990593	other superficial source	1999	Taiwan, China	0.5	S	25% (1/4)
	817	F2017a006	blood	2017	Taiwan, China	1	S	
	839	F2017f045	blood	2017	Taiwan, China	1	S	
	854	CT217	—	2013	Taiwan, China	32	R	
CC16	4	J930949	sputum/BAL	1993	Frankfurt, Germany	1	S	0 (0/3)
	47	b30642/4/04	other sterile site	2004	UK	2	S	
	52	b30451/4/04	other sterile site	2004	UK	0.25	S	
CC17	214	T08	sputum/BAL	—	India	4	I	100% (6/6)
		T17	sputum/BAL			32	R	
		T28	sputum/BAL			>64	R	
		T29	sputum/BAL			64	R	
	215	T13	sputum/BAL	—	India	>64	R	
	216	T26	sputum/BAL	—	India	32	R	
CC18	222	T14	sputum/BAL	—	India	>64	R	66.7% (2/3)
	223	T42	sputum/BAL	—	India	2	S	
	224	T41	sputum/BAL	—	India	4	I	
CC19	330	CTR-11	Urine	2019	Wuhan, China	0.5	S	0 (0/5)
	333	IRCCS 3	blood	2015	Sicily, Italy	<=1	S	
		IRCCS 42	blood	2017		<=1	S	
		IRCCS 43	blood			<=1	S	
	532	618	urine	2014	Shanghai, China	1	S	
CC20	183	YM060559	blood	2006	Taiwan, China	>64	R	50% (2/4)
		NHUE37	—	2007		>64	R	
	517	598	faeces	2013	Shanghai, China	2	S	
	831	F2017d002	blood	2017	Taiwan, China	2	S	
CC21	575	YM140019	urine	2014	Taiwan, China	4	I	100% (3/3)
	601	YFA120295	—	2012	Taiwan, China	8	R	
	819	F2017a016	blood	2017	Taiwan, China	64	R	
CC22	343	CTR-14	Vaginal swab	2019	Wuhan, China	1	S	0 (0/4)
		CTR-80	Urine			0.25	S	
	723	CTR-30	Vaginal swab	2019	Wuhan, China	0.125	S	
	998	CTR-44	Vaginal swab	2019	Wuhan, China	0.25	S	
CC23	747	IRCCS 1	blood	2015	Sicily, Italy	<=1	S	0 (0/13)
		IRCCS 2	—			<=1	S	
		IRCCS 15	blood	2016		<=1	S	
		IRCCS 23	blood			<=1	S	
		IRCCS 24	—			<=1	S	
		IRCCS 31	—			<=1	S	
		IRCCS 32	—			<=1	S	
		IRCCS 37	blood	2017		<=1	S	
		IRCCS 39	—			<=1	S	
	748	IRCCS 7	blood	2015	Sicily, Italy	<=1	S	
		IRCCS 8	—			<=1	S	
	759	IRCCS 51	blood	2015	Sicily, Italy	<=1	S	

		IRCCS 52	—			<=1	**S**	
**CC24**	**508**	587	urine	2013	Shanghai, China	8	**R**	**100%** (5/5)
		588	urine	2014		16	**R**	
		589	urine	2015		16	**R**	
	**851**	CT152	—	2014	Taiwan, China	16	**R**	
	**892**	CTS13	blood	2015	Thailand	64	**R**	
**CC25**	**665**	YM140912	blood	2014	Taiwan, China	0.5	**S**	**0** (0/3)
	**781**	CT268	—	2012	Taiwan, China	2	**S**	
	**910**	YM140730	faeces	2014	Taiwan, China	0.25	**S**	

435 isolates with fluconazole MICs obtained from the C. tropicalis MLST database (https://pubmlst.org/ctropicalis) and 87 isolates from Wuhan were analyzed. The information of C. tropicalis isolates in CC1~CC25 was listed in the table. FNS CCs are labeled in red (CC1, CC2, CC4, CC11, CC14, CC17, CC21, CC24), and FS CCs are labeled in blue (CC8, CC10, CC16, CC19, CC22, CC23, and CC25). The FNS rate greater than 80% is marked as red and less than 10% is marked as blue. R (fluconazole resistant) and I (fluconazole intermediate) are labeled in red, and S (fluconazole susceptible) is labeled in blue. —, not available.
