# Supplementary material for: Multilocus Sequence Typing Reveals Clonality of Fluconazole-Nonsusceptible Candida tropicalis: A Study From Wuhan to the Global
Source: Front Microbiol. 2020 Nov 17;11:554249. doi: 10.3389/fmicb.2020.554249 (PMC7705220; doi:10.3389/fmicb.2020.554249)
Supplement: Supplementary file 3 [file Table_3.PDF]

Supplementary Table S3 Summary of *Candida tropicalis* isolates of DST225, DST375, DST506, and DST 546 from previous studies*

DST	year	Location	Isolate number	Source	FLC MIC (μg/mL)	References
225	2014-2015	Beijing,China	Ct01R Ct07R Ct08R Ct09R	Clinical	64 ~ >256	1
	2012	Taiwan, China	F85	Environment	4	2
	2012-2016	Taiwan, China	F2012i031 F2013g040 F2014g069 F2015f002 F2015f066 F2016a011 F2016a073 F2016d089 F2016e086	Clinical	32 ~ 512	3
376	2013-2017	Taiwan, China	F2012f046 F2012f083 F2013a013 F2015c056 F2017c014 F2017c078	Clinical	64 ~ 512	3
	2012-2015	Shanghai, China	RC51 RC165 RC181 RC193 RC289 RC366 RC519 RC527	Clinical	16 ~168	4
506	2014-2015	Taiwan, China	F2014f079 F2014 g052 F2015a060 F2015d058 F2015e040 F2015e098	Clinical	256 ~ 512	3
546	2014	Taiwan, China	F2014f003	Clinical	—	5

** DST225, DST375, DST506, and DST 546 belong to Wuhan local CC2 in this study. —, not available.*

References

1. Jin L, Cao Z, Wang Q, Wang Y, Wang X, Chen H, et al. 2018. MDR1 overexpression combined with ERG11 mutations induce high-level fluconazole resistance in *Candida tropicalis* clinical isolates. BMC Infect Dis. 18:162.
2. Lo HJ, Tsai SH, Chu WL, Chen YZ, Zhou ZL, Chen HF, et al. 2017. Fruits as the vehicle of drug resistant pathogenic yeasts. J Infect. 75:254-262.
3. Chen PY, Chuang YC, Wu UI, Sun HY, Wang JT, Sheng WH, Lo HJ, Wang HY, Chen YC, Chang SC. 2019. Clonality of Fluconazole-Nonsusceptible *Candida tropicalis* in Bloodstream Infections, Taiwan, 2011-2017. Emerg Infect Dis. 25:1660-1667.
4. Wang Y, Shi C, Liu JY, Li WJ, Zhao Y, Xiang MJ. Multilocus sequence typing of *Candida tropicalis* shows clonal cluster enrichment in azole-resistant isolates from patients in Shanghai, China. Infect Genet Evol. 2016;44:418-424.
5. Jolly K. *Candida tropicalis* MLST central database sited at the University of Oxford. https://pubmlst.org/ctropicalis
